# Biological Stress Reactivity and Introspective Sensitivity: An Exploratory Study

**DOI:** 10.3389/fpsyg.2020.00543

**Published:** 2020-03-26

**Authors:** Mauricio Barrientos, Leonel Tapia, Jaime R. Silva, Gabriel Reyes

**Affiliations:** ^1^Centro de Apego y Regulación Emocional, Facultad de Psicología, Universidad del Desarrollo, Santiago, Chile; ^2^Centro de Estudios en Neurociencia Humana y Neuropsicología, Facultad de Psicología, Universidad Diego Portales, Santiago, Chile

**Keywords:** introspection, biological stress reactivity, TSST, consciousness, cortisol

## Abstract

Reaction to stressful events has an impact on several cognitive processes. High levels of stress can be detrimental to working memory, attention and decision-making. Here, we investigated whether individuals’ reactivity to stress is related to their introspective sensitivity (i.e., how well individuals monitor their own cognitive processes). To this aim, 27 participants (16 women, mean 20 years old) were exposed to a psychosocial stress protocol (trier social stress test, TSST), where individuals were asked to simulate a job interview and perform arithmetic calculations in front of a panel of experts. The salivary cortisol concentration, which is considered a hormonal index of stress reactivity, was collected during the TSST through the enzyme immunoassay DRG cortisol ELISA kit. Based on literature recommendations, we classified participants as responders and non-responders to the TSST. In a second session, through a visual search paradigm, we evaluated the introspective sensitivity of the participants. We evaluated how these individuals (i) monitor their own performance (through a confidence estimation), (ii) monitor their own attentional shifts (through a subjective number of scanned items estimation, SNSI), and (iii) monitor their own response times (through an introspective response time estimation, iRT). We found that individuals with lower biological reactivity to stress are more accurate in estimating their SNSI (*p* = 0.033) and iRT (*p* = 0.002), and in evaluating their own performance (*p* = 0.038) through their confidence. We argue that the effect of stress on introspection is not limited to a particular type of introspective evaluation, but rather consists of a general alteration of the introspective mechanism.

## Introduction

Introspection refers to the ability to access and report one’s own mental content (Flavell, 1979). In everyday life, it is natural for individuals to report knowing the time between one decision and another (Corallo et al., 2008), feeling the effort involved in executing some decisions (Naccache et al., 2005), and/or knowing the level of confidence associated with such decisions (Koriat, 2012). All these cases denote that individuals evaluate their own mental contents. Cognitive scientists suggest that this ability, known as introspection (or metacognition), is a key mechanism for controlling one’s behavior (Nelson and Narens, 1990) and for social interactions (Shea et al., 2014). In experimental psychology, introspection is investigated through its sensitivity. That is, in the context of signal detection theory (Galvin et al., 2003), researchers investigate how accurate individuals are to detect objective aspects of their decisions (e.g., the [objective] decision time elapsed between stimulus presentation and the perceptual decision) from [subjective] estimates from visual analog scales (introspective response times, iRT; Corallo et al., 2008; Marti et al., 2010). Critically, the study of the introspective judgments sensitivity may incur in different formats, depending on the introspective question. In this line, it is possible to investigate how accurate an individual is in subjectively monitoring their correct *vs*. incorrect decisions from confidence judgments (rev. Fleming and Frith, 2014). Other studies focus their interest on the introspection of the attentional shifts (subjective number of scanned items, SNSI; Reyes and Sackur, 2014, 2017, 2018; Gajdos et al., 2019). In cognitive sciences, there is no consensus of whether there is a common introspective mechanism for all these cases, or if different subjective estimates involve different psychological processes; neither if external factors (e.g., pharmacological manipulation of neuromodulators, Hauser et al., 2017) that alter a certain introspective judgment will also alter other introspective judgments.

Recent research into experimental introspection has been interested in the role of metacognition in psychiatric disorders (Rouault et al., 2018). In this vein, our previous studies (Reyes et al., 2015) determined that biological reactivity to a psychosocial stressor is a determinant of how efficient individuals are at monitoring their decisions through confidence estimates: the more sensitive an individual is to stress, the worse introspective sensitivity is evidenced. Here, our main aim is to expand the results of Reyes et al. (2015) to three different introspective dimensions: confidence in the decision, introspection of the decision time (iRT) and the estimation of attentional shifts (SNSI). We hypothesize that the effect of stress reactivity on introspective sensitivity, operationalized through confidence judgments, will also be observed in the introspective sensitivity of subjective time (iRT) and attentional (SNSI) judgments.

It is well-known that stressful situations impact a variety of cognitive processes (Otto et al., 2013) by affecting central executive resources (Lupien et al., 2007). High levels of stress can be detrimental to working memory (Vedhara et al., 2000; Matthews and Campbell, 2010), visual attention (Sänger et al., 2014), decision-making (Porcelli and Delgado, 2009; Starcke and Brand, 2012; Rued et al., 2019), and also to higher-order cognitive processes (Sliwinski et al., 2006; Schwabe and Wolf, 2011). At the physiological level, stress leads to a cascade of neuromodulator production, all of which impacts brain functions, with a release of catecholamines (noradrenaline, dopamine and then adrenaline) and cortisol response (Sapolsky et al., 2000; Hermans et al., 2014). These endocrinal changes prepare the body for “fight or flight,” enhancing amygdala function and disadvantaging the allocation of cognitive resources in specific cortical areas (dorsolateral and medial prefrontal cortex; see Arnsten, 2009; Hermans et al., 2014) associated with endogenous attention and high-order cognitive processing (Fleck et al., 2005; Rounis et al., 2010; Fleming and Dolan, 2012). According to this, the stress effect on high-order functions should have a general impact on the capacity for introspection, assuming that introspection operates through a supramodal and unified mechanism (Faivre et al., 2018).

In the first session, we applied an interpersonal stress induction protocol: the trier social stress test (TSST; Kirschbaum et al., 1993). The participants were asked to perform a 5-min speech and mental arithmetic in front of a group of non-supportive judges, with cortisol samples taken before and after the stress induction. According to their hormonal responses (i.e., the variation in salivary cortisol concentration during the TSST), we classified participants as responders (R) and non-responders (NR), following literature recommendation (Miller et al., 2013). In the second session (1 week later), we investigated the introspective sensitivity, understood as the accuracy of subjective estimates of participants’ own performance in a visual search task. During the visual search paradigm protocol, participants were asked to report three introspective visual analog scales – the SNSI scale (Reyes and Sackur, 2014, 2017, 2018; Gajdos et al., 2019), the iRT scale (Corallo et al., 2008; Marti et al., 2010) and a confidence scale (e.g., Fleming et al., 2010) – after each trial. We reasoned that these three scales would help us delineate introspective profiles. According to the cognitive science literature on the stress effect on high-order processes (Lupien et al., 2007), we predicted that across these three introspective scales, the NR group should evidence better introspective sensitivity than the R group, and that this difference would not be explained by differences in perceptual performance.

## Materials and Methods

### Participants

Twenty-seven undergraduates (20.4 ± 1.7 years old; 16 women) were evaluated in two sessions. Exclusion criteria were: a body mass index <18 or >30 kg/m^2^; receiving medical treatment known to affect the hypothalamus-pituitary-adrenal axis; a history of psychiatric or neurological disorders; abnormal vision; smokers; pregnant women and women taking oral contraceptives. Participants were asked not to eat or brush their teeth 1 hour before the TSST, and to not drink alcohol or play sports the day before. This study was approved by the Ethics Committee of the Universidad del Desarrollo. All subjects gave written informed consent in accordance with the Declaration of Helsinki. Participants were compensated USD$10 for both sessions. The compensation was received at the end of the second session. Experimental sessions were scheduled from 2:30 to 6:30 PM. A week later, the same participants were recruited to perform the visual search task.

### Stimuli and Procedure

#### Session 1

##### Trier social stress test

**FIGURE 1:**
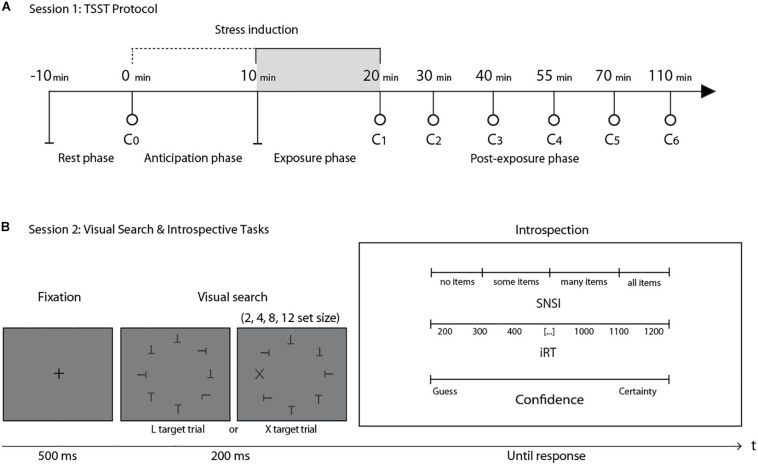
**(A)** General structure of session 1. After a 10-min rest, the first saliva sample (C0) was taken as a baseline measure. C1 was taken immediately after a 20-min stress induction protocol (TSST). The next five cortisol measures (C2–C6) were taken in the following 90 min after stress induction. **(B)** General structure of the visual search task in session 2. After a fixation cross, participants were presented with one of two possible conditions: finding an L among a set of Ts or finding an X among a set of Ts. After the perceptual decision, three introspective scales were presented simultaneously: the SNSI scale, the iRT scale and a confidence scale. This session took place one week after session 1. *N* = 27.

We applied the TSST (Kirschbaum et al., 1993), with seven interspersed saliva samples to assay cortisol concentration (Figure 1A). The experimental literature has reported that salivary cortisol levels are particularly sensitive to this standardized protocol, showing a cortisol increase after 20 min of stress induction (Allen et al., 2014; Goodman et al., 2017; Liu et al., 2017). According to the TSST protocol, participants were asked to rest in a room for 10 min after arriving to the laboratory. After this period, at time 0, a research assistant, with no knowledge of the objectives of the experiment, extracted the first saliva sample (C0). Afterward, participants were taken to another room were the TSST took place. For this test, participants had to simulate a job interview. They were given 10 min alone to prepare a 5-min speech. The speech was performed at time + 10 min in front of a “selection committee” composed of three non-supportive judges of the same gender as the participant. After the speech, the committee asked the participants to perform mental arithmetic during 5 min. Both the presentation and the arithmetic task were videotaped. At time +20 min, the committee closed the interview and the participants returned to the first room, where the assistant took the second saliva sample (C1). In this room, the participants rested for 90 min, while five more saliva samples were taken at +30, +40, +55, +70, and +110 min (Figure 1A). Salivary cortisol was measured with the enzyme immunoassay DRG cortisol ELISA kit according to the manufacturer’s specifications (SALI-TUBES: SLV-4158, see Supplementary Material I). Based on the participants’ stress response to the TSST (Miller et al., 2013), participants were classified as Responders (*N* = 15; age = 20.21 ± 1.53; 7 women) and Non-Responders (*N* = 12; age = 20.67 ± 1.92; 9 women).

#### Session 2

##### Visual search task

Stimuli consisted of a set of black letters (T, L, or X, size: 0.8° × 0.6°, luminance: 0.59 cd/m^2^) on a uniform gray background (luminance: 44.1 cd/m^2^), presented on an imaginary circle (radius: 6.2°) around a central fixation spot at the center of the screen. Individual orientation for each letter was randomized (0°, 90°, 180°, and 270°). Stimuli were equally spaced on the imaginary circle, while their overall orientation was randomized for each trial. Stimuli were presented on a CRT screen (size 17″, resolution of 1024 × 768 pixels, refresh rate of 100 Hz, viewing distance ∼55 cm). The experiment took place in a dark room. Stimuli were presented for 200 ms, preceded by a fixation spot presented for 500 ms. Participants were instructed to decide on the presence or absence of a target (L or X) within the set of distractors (Ts), by pressing the “Q” or “W” key on a standard Spanish “QWERTY” keyboard. Half of the trials were target-absent trials. Target-present trials contained one “L” or one “X.” Set-size (2, 4, 8, or 12 items) and the presence-absence of a target were fully crossed. X targets were meant to create easy, “pop-out” searches, while L targets were introduced to create difficult, attentional searches. Immediately after the response, three continuous introspective scales were simultaneously presented: (i) SNSI (though this scale registered numerical scores from 0 to 100 in steps of 1, it was labeled with four qualitative categories: “no items,” “some items,” “many items,” and “all items”): how many items were scanned before the target was identified; (ii) iRT (from 200 to 1200 ms, labeled in steps of 100): how much time elapsed between stimulus presentation and perceptual decision; (iii) confidence (from 0 to 10 in steps of 1, with labels “Guess” and “Certainty” at both ends of the scale): the level of confidence associated with the correctness of the perceptual decision (Figure 1B). The experimental session comprised 256 trials divided into 8 blocks, with a 60-s pause between each. Before beginning the experiment, participants had 32 training trials.

### Statistical Analysis

We implemented multiple and independent linear mixed model (LMMs) analyses and *t*-tests as appropriate. Statistical analyses were performed with SPSS-23. Regarding cortisol analysis (Session 1) and following recommendations in the literature, baseline-to-peak cortisol increases (max cortisol-peak [C2-C6] minus baseline [C0]) were calculated to classify the participants as Responders [R] or Non-Responders [NR] (Miller et al., 2013; Supplementary Material I). Participants with a baseline-to-peak cortisol increase above/below 1.5 nmol/l were categorized as Responders (*M* = 3.89; SE = 0.25 nmol/l; range: [2.4, 6.3]; *N* = 15) and Non-Responders (*M* = −0.03; SE = 0.21 nmol/l; range: [−1.1, 1]; *N* = 12), respectively. After that, the cortisol scores were Box-Cox transformed individually in order to normalize them. We followed methodological recommendations for longitudinal cortisol samples after stress induction (Miller and Plessow, 2013) and applied the following correction:

$$c^{\prime }=\frac{c^{0.26}-1}{0.26}$$

where *c* are individual cortisol measures. All subsequent analyses were done on the normalized data. Additionally, in order to further investigate the differences in cortisol response, an indicator related to cortisol production during the task was calculated: the area under the curve with respect to the increases (AUCi). This value measures the total variation of cortisol during the experimental protocol compared to a baseline, and is calculated by the formula presented in Pruessner et al. (2003) as:

$${\text{AUC}}_{i}=\left(\sum_{i=1}^{n}\frac{\left(c_{i}^{\prime }+c_{i-1}^{\prime }\right)\times \Delta \,t_{i}}{2}\right)-c_{0}^{\prime }\,\sum_{i=1}^{n}\Delta \,t_{i}$$

where *c*′ represents normalized cortisol scores in sample *i*, and Δ*t*_*i*_ represents the time between cortisol sample *i* and *i* − 1 in minutes.

Regarding the visual search task (Session 2), individual trials with response times (RTs) below 200 ms and above 2000 ms were excluded from all analyses (6.7% of the trials). In order to account for individual differences, all analyses were done on individual means for each experimental condition – eight mean points per participant (Set-Size [4] × Search Type [2]) – rather than on a trial-by-trial basis, unless otherwise stated. In order to present a unified performance index that controlled for the speed-accuracy trade-off, we calculated linear integrated speed-accuracy scores (LISAS: Vandierendonck, 2017) and confirmed through Balanced Integration Score (BIS: Liesefeld and Janczyk, 2019). On the one hand, we choose LISAS because of the high amount of conditions with an error rate equals zero. LISAS were calculated by the following formula:

$$\text{LISAS}={\text{RT}}_{\text{c}}+\text{PE}\times \frac{S_{\text{RT}}}{S_{\text{PE}}}$$

where RT_c_ is the mean response time in correct trials, PE is the proportion of error, and *S*_RT_ and *S*_PE_, their standard deviations, respectively. Lower LISAS values denote better performance. On the other hand, we choose BIS because it gives equal weights to both speed and accuracy. We calculated BIS following the formula:

$$\text{BIS}=Z_{\text{PC}}-Z_{\text{RTC}}$$

with BIS being equal to the difference between the standardized means of correct responses (*Z*_PC_) and response time in correct trials (*Z*_RTC_). Higher BIS values denote better performance. Then, regarding the SNSI scale, we calculated how much participants misjudge the number of items scanned during the task through a model proposed by Gajdos et al. (2019). This model allowed us to calculate the bias in the responses to the SNSI scale of each participant through the following formula:

$$\left|{\text{SNSI}}_{\text{error}}\,\left(x\right)\right|$$

$$=\frac{\left({\text{SNSI}}_{n}\,\left(x\right)-1\right)\,\left(n-m\right)-\left(n-1\right)\,\left({\text{SNSI}}_{n}\,\left(x\right)-{\text{SNSI}}_{m}\,\left(x\right)\right)}{n-m}$$

with SNSI*_*i*_*(*x*), the mean SNSI for set-size *i* and search type *x*, and *n* and *m* being different set-sizes utilized during the task. During our analyses, we used *n* = 2 and *m* = 12 to compute SNSI_error_. Under the assumptions of the model, this value allowed us to estimate the mismatch between a theoretical estimation and the SNSI. Higher values denote a larger mismatch -or bias. For the confidence scale and the estimation of decision time, we evaluated the correlation between the confidence scores and the performance in the visual search task, and between the iRTs and the objective decision time (RTs), respectively. Finally, for the introspective scales’ analyses, all *p*-values were Bonferroni corrected to account for the three simultaneously collected dependent variables.

## Results

### Session 1

#### Biological Stress Reactivity

**FIGURE 2:**
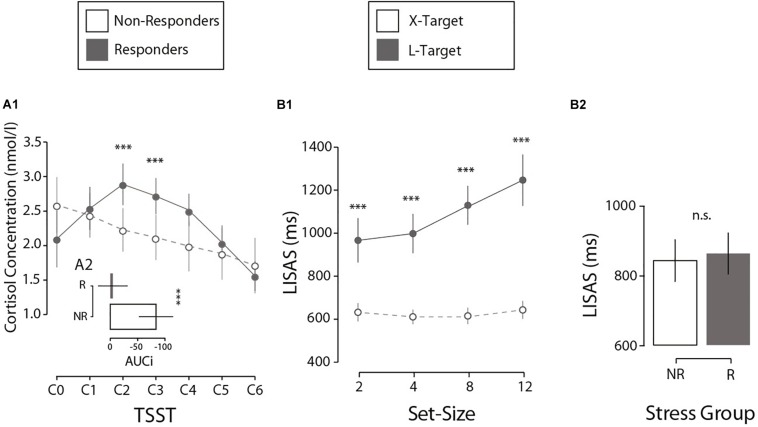
**(A1)** Cortisol concentration as a function of the TSST phase for both experimental groups. Error bars here and in the following analysis denote ±2 SE. **(A2)** Differences between experimental groups in cortisol production (AUCi) during the first session. **(B1)** Interaction between set-size and search type in perceptual performance (LISAS) during the second session. **(B2)** Comparison of performance by stress group, measured by LISAS, during the second session. *N* = 27. ***p < 0.001, ^n.s.^*p* > 0.05.

First, we analyzed how the salivary cortisol varies throughout the TSST protocol. The results indicate that the TSST induces a differential variation in the biological response to stress across participants (salivary cortisol concentration, Figure 2A1). First, through an LMM analysis, we observed a quadratic effect of Cortisol Sample (seven samples: from C0 to C6), a main effect of Stress Group (NR vs. R) and their interaction on normalized saliva cortisol scores (*M* = 2.25; SE = 0.05 nmol/l). A participants’ random intercept was added to the model. Results demonstrated a significant quadratic effect of Cortisol Sample (*F*(1, 159) = 49.42, *p* < 0.001, β = −0.06), with an interaction between Cortisol Sample × Stress Group (*F*(2, 159) = 13.37, *p* < 0.001, β = −0.05). A deeper inspection revealed a quadratic effect on the R group (*F*(1, 88) = 123.69, *p* < 0.001, β = −0.11), with no variation for the NR group (*p* = 0.664). In summary, the R group presented a quadratic cortisol modulation with a peak at a specific time (C2, 10 min after stress induction, Figure 2A1) according to the stress literature (Rimmele et al., 2007; Petrowski et al., 2010; Allen et al., 2014). Crucially, there were no differences in the baseline cortisol concentration (C0) between the two groups (*p* = 0.106), suggesting that variation in cortisol cannot be explained by individual differences. In addition, to confirm the differences between the Stress Groups, we calculated the total variation in cortisol produced during the task compared to the individual baseline (AUCi; Pruessner et al., 2003). Independent *t*-tests on AUCi (*M* = −39.65; SE = 12.60 nmol/l per min) showed that the R and NR groups differed significantly in cortisol production (Δ*M* = 78.94 nmol/l per min, *t*(25) = 3.856, *p* < 0.001, Cohen’s *d* = 1.49, Figure 2A2). A control analysis showed no differences in baseline-to-peak cortisol increases by gender (*p* = 0.169) or age (*p* = 0.566). In summary, these results confirm that the two groups differs in their hormonal reactivity to stress.

### Session 2

#### Perceptual Performance Results (LISAs, RTs, Error Rates)

Perceptual performance during the visual search task was investigated. Results showed a traditionally reported interaction in visual search literature (Treisman and Gelade, 1980; Wolfe, 1994) between Search Type (X, L) and Set-Size (2, 4, 8, 12; Figure 2B1). Crucially this interaction pattern did not differ when considering Stress Group (NR vs. R). First, an LMM analysis was run on LISAS (*M* = 855, SE = 21.3) on present-target trials with the factors Search Type (X, L), Set-Size (2, 4, 8, 12), Stress Group (NR, R) and their interactions. A participants’ random intercept was included in the model. The analysis showed a significant effect for Search Type (*F*(1, 183) = 51.4, *p* < 0.001, β = 313.8), Set-Size (*F*(1, 183) = 36.3, *p* < 0.001, β = 25.2), and the Search Type × Set-Size interaction (*F*(1, 183) = 29.5, *p* < 0.001, β = 23.7). All other effects reported no significant results (all *p*s > 0.353). Critically, no effect for Stress Group was found (*p* = 0.471), suggesting that perceptual performance was not affected by stress reactivity (Figure 2B2). *Post hoc* comparisons showed differences in LISAS between X and L for each Set-Size (2 items: Δ*M* = −334.5 ms, *t*(34.3) = −6.01, *p* < 0.001, *d* = 1.64; 4 items: Δ*M* = −387.2 ms, *t*(33.1) = −7.98, *p* < 0.001, *d* = 2.17; 8 items: Δ*M* = −514.4 ms, *t*(35.1) = −10.5, *p* < 0.001, *d* = 2.87; 12 items: Δ*M* = −603.4 ms, *t*(32.1) = −9.57, *p* < 0.001, *d* = 2.61).^1^ An independent analysis on mean RTs (*M* = 788, SE = 16.37 ms) and mean error rate (*M* = 0.13; SE = 0.013%) confirmed no significant differences in performance between Stress groups (all *p*s > 0.51), as expected. In short, these results confirm no effect of the Stress Group on objective performance. Our interest now is to investigate an exclusive effect on introspective performance.

#### Introspective Performance Results

##### Subjective number of scanned items

**FIGURE 3:**
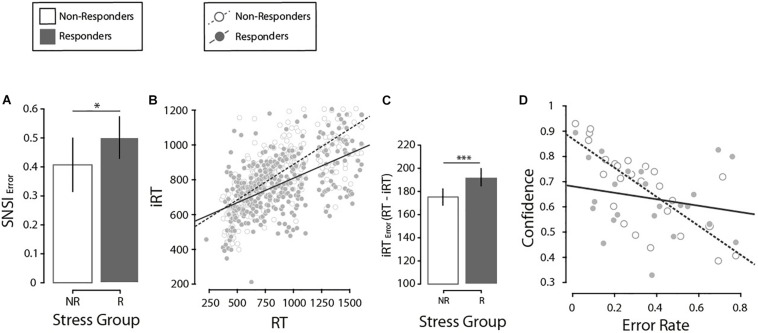
**(A)** Comparison of the misjudgments in the number of items revised (SNSI Error), by stress group. Error bars here and in the following analysis denote ±2 SE. **(B)** Regression of the introspective response time and the response time, by stress groups. **(C)** Comparison of the error in the estimation of the response times (iRT Error, calculated as the absolute value of RT minus iRT) by stress groups. **(D)** Regression of confidence and error rate, by stress groups. *N* = 27. *p < 0.05, ***p < 0.001.

Next, we investigated differences in SNSI. On a trial-by-trial basis, we calculated SNSI adjusted for Set-Size to obtain a real estimate of the number of items scanned. An LMM was run on SNSI in correct present-target trials (*M* = 3.23, SE = 0.18 items). Fixed effects of Search Type (X, L), Set-Size (2, 4, 8, 12), Stress Group (NR, R) and all possible interactions were investigated, and a participants’ random intercept was included in the model. Results indicated a significant main effect of Set-Size (*F*(1, 176) = 513.4, *p* < 0.001, β = 0.44) and its interaction with Stress Group (*F*(1, 176) = 9.1, *p* = 0.021, β = 0.13). No other significant effects were found (all *p*s > 0.679). Closer inspection on the interaction between Set-Size and Stress Group showed that both the R and NR groups presented a positive and significant relationship between SNSI and Set-Size, but this was higher in the NR group (Δβ = 0.10, *t*(180) = 3.04, *p* = 0.020), suggesting a better fit in the NR group based on the increase in Set-Size compared to the R group. Next, in order to further analyze the accuracy with which participants estimate the number of items they scanned (SNSI), we calculated an estimation for how much participants misjudge this number (SNSI_error_) through a rationale proposed by Gajdos et al. (2019). We thank an reviewer for the suggestion of implementing this additional analysis. First, one participant (with >0.93 SNSI_error_) was excluded from this analysis. Then, we calculated two values of SNSI_error_ for each participant – one for each search type. Finally, we ran a two-way ANOVA on SNSI_error_ with Search Type (X, L) and Stress Group (NR, R) as fixed factors. We found a significant effect of Stress Group, with a higher error in the R group than the NR group (Δ*M* = 0.12, *F*(1, 47) = 4.86, *p* = 0.033, η^2^*_*p*_* = 0.090, Figure 3A). This suggests an effect of stress on how accurately participants estimated the number of items scanned: the R group showed a higher SNSI_error_ than the NR group.

##### Introspective response times

Next, we analyzed how well participants monitored their own RTs through an iRT estimation on a trial-by-trial basis. Results indicated that participants that responded to psychosocial stress (i.e., the R group) were less sensitive in tracking their own RTs than individuals from the NR group; this difference was stronger in the L-target than the X-target. First, we searched for potentially influential points in the regression analysis [i.e., outlier points with both a standardized residual higher than 2 and a leverage value above the criterion proposed by Cohen et al. (2003)] and excluded 4.6% of trials. Then, an LMM was run on correct present-target trials (iRT: *M* = 747.29, SE = 3.76 ms), with fixed effects of RT (*M* = 710, SE = 4.19 ms), Search Type (X, L), Set-Size (2, 4, 8, 12), Stress Group (NR, R) and all possible interactions. In addition, a participants’ random intercept was included in the model. Significant effects of RT (*F*(1, 2722.4) = 359.19, *p* < 0.001, β = 0.15), Search Type (*F*(1, 2711) = 52.73, *p* < 0.001, β = −161.27) and Set-Size (*F*(1, 2706.4) = 3.72, *p* = 0.011, β = −91.67) were noted. We also found significant interactions between RT × Search Type (*F*(1, 2713.9) = 34.62, *p* < 0.001, β = 0.17), and RT × Stress Group (*F*(1, 2722.4) = 4.2, *p* = 0.041, β = 0.07, Supplementary Material II for interaction figures). Crucially, this last interaction suggests that the regression coefficient between RT and iRT was particularly different depending on the Stress Group. In fact, even though a closer inspection demonstrated that both stress groups presented a significant and positive relationship (NR: β = 0.36, *p* < 0.001; R: β = 0.31, *p* < 0.001), a *post hoc* comparison showed differences between them (Δβ = 0.05, *t*(2742.6) = 2.19, *p* = 0.028), suggesting that compared to the NR group, participants from the R group were less accurate in tracking the variability of their own RTs (Figure 3B). In this line, the NR group showed a lower absolute distance between iRT and RT (Δ*M* = 17.09 ms, *F*(1, 2759) = 10.68, *p* = 0.002, Figure 3C). All this suggests again that the NR group presented a time estimation more accurate than the R group.

##### Confidence

Finally, we investigated how participants monitor their own correct and incorrect responses from confidence estimations. Following introspection literature (e.g., Son and Metcalfe, 2000), we used Goodman and Kruskal’s gamma correlation to determine the association between Error rate (*M* = 0.13, SE = 0.006) and Confidence (*M* = 8.16, SE = 0.04) in present-target trials. As expected, we found a negative correlation between Error rate and Confidence (γ = −0.83, ASE = 0.014, *p* < 0.001). Next, in order to ascertain how participants’ stress response impacted the relationship between Confidence and Error rates, we implemented an LMM analysis on confidence ratings in present-target trials. The effects of Error rate (correct and incorrect), Stress Group (NR, R) and their interaction as fixed effects were investigated. Also, a participants’ random intercept was added to the model. There was evidence of a main effect of Error rate (*F*(1, 195.6) = 442.2, *p* < 0.001, β = −6.12) and the interaction Stress Group × Error rate (*F*(1, 195.7) = 4.4, *p* = 0.038, β = −1.35). Closer inspection showed a higher relationship with Error rate in the NR group (Δβ = 1.36, *t*(195.7) = 2.09, *p* = 0.038, Figure 3D). These results suggest again that, although there is a general relation between Error rates and Confidence estimates, participants in the NR group were more accurate at monitoring their own performance (in terms of correct/incorrect responses) in the visual search paradigm.^2^ Results in confidence estimate are in line with those reported for SNSI and for iRT, as expected. The NR group presented greater introspective sensitivity than the R group. Importantly, we investigated whether these introspective indexes could be correlated, which could explain the similar pattern described. Three independent LMMs (one for each introspective scale) revealed that this is not the case (all *p*s > 0.17), meaning all three scales account for different aspects of introspection.

## Discussion

The aim of this study was to explore if the biological reactivity to stress has an impact on three ways to assess introspective sensitivity (high order processes): the ability of individuals to subjectively monitor objective aspects of their decisions. We determined differences in reactivity to stress through the increase in salivary cortisol concentration after a stressful event (TSST protocol, Kirschbaum et al., 1993). Introspective sensitivity was operationalized (i) by the subjective estimation of the participant’s own attentional shifts (SNSI); (ii) by the subjective estimation of response times (iRT); and (iii) by the resolution of confidence judgments. Previous research indicate that individuals with hormonal reactivity (cortisol concentration in saliva, Reyes et al., 2015) to stressful contexts, are associated with less capacity to monitor their own correct and incorrect responses through confidence judgments. In this exploratory study, we found that this effect is also present when individuals monitor their own decision times (iRT), and when they try to describe their attentional shifts during perceptual decisions (SNSI). In other words, this study shows that hormonal reactivity to stressful contexts is associated with low introspective sensitivity profile. Critically, here we evidenced no differences in perceptual performance (LISAS, RTs, or Error Rate) between stress reactivity groups. Thus, the differences observed in introspection are due not to a trivial link between perceptual and introspective performance (i.e., the poorer the performance at the primary perceptual level, the less information there is for the introspective task, cf., Galvin et al., 2003), but to specific introspective differences between the stress groups.

Differences in stress reactivity could be associated to a global impact on the higher order executive functions (Hermans et al., 2014). Thus, this well-known hormonal effect on dorsolateral and medial prefrontal cortex (Arnsten, 2009), could interact with the human introspective system. More research is necessary to formulate precise hypotheses of how the neuro-hormonal system impacts introspection. We can speculate that stress alters the ability of individuals to direct their attention toward relevant information to build their introspective judgments. However, if this is the case we should observe some impact on the task performance (e.g., response times, error rate). Another possibility is that the effect of stress promotes states of mental rumination, which negatively and exclusively impacts the participants attention during the introspective process. This could be investigated through the time that participants use to respond to each of these introspective scales. It could even be the case that hormonal reactivity introduces new pieces of interoceptive information (e.g., emotional states related to anxiety and stress), which modify the way in which introspective judgments are computed. In summary, more research is needed to clarify the stress effect here reported.

Finally, it is necessary to establish some considerations. In our exploratory study, stress induction was carried out 1 week prior to the introspective session. Consequently, our results should be interpreted as an association between the individual introspective profile and its neuro-hormonal response to stress. Future studies should confirm that during the execution of the introspective scales, individuals show levels of biological stress reactivity comparable to those evidenced in session 1. In the same line, future studies could implement pharmacological stressors during the execution of these scales. On the other hand, our results suggest the presence of a common psychological mechanism underlying introspective processes across these three subjective scales. Finally, methodological considerations on the scales could be introduced to specify our introspective estimation. For instance, the estimation of SNSI misjudgment (SNSI_error_), can be improved incorporating eye-tracking measures during the visual search protocol (e.g., Reyes and Sackur, 2014; Marti et al., 2015). Despite all this, our exploratory study can conclude that normative biological reactivity to stress is associated with a systematic decrease in introspective sensitivity, not only in reports of confidence in the decision, as the literature has previously reported (Reyes et al., 2015), but also in other two introspective scales. From this we can hypothesize the presence of a common or shared mechanism between different introspective domains.

## Data Availability Statement

The datasets generated for this study are available on request to the corresponding author.

## Ethics Statement

The studies involving human participants were reviewed and approved by the Comité de Ética Institucional en Investigación, Universidad del Desarrollo. The patients/participants provided their written informed consent to participate in this study.

## Author Contributions

GR and JS: conceptualization, methodology, software, and supervision. GR, LT, and MB: analysis, visualization and writing.

## Conflict of Interest

The authors declare that the research was conducted in the absence of any commercial or financial relationships that could be construed as a potential conflict of interest.

## Footnotes

1 The same results were evidenced with the Balanced Integration Score (BIS; Liesefeld and Janczyk, 2019). A main effects of Search Type (*F*(1, 183) = 49.9, *p* < 0.001, Δβ = 1.6), Set-Size (*F*(1, 183) = 100.9, *p* < 0.001, Δβ = −0.19) and their interaction (*F*(1, 183) = 47.1, *p* < 0.001, Δβ = 0.18) were observed. All others effects reported no significant results (all ps > 0.442). These results confirmed the patterns observed in the analysis of LISAS.

2 Additionally, we calculated metacognitive efficiency scores (*meta-d′/d′*; Fleming and Lau, 2014) for each participant. Independent groups *t*-tests showed no differences between Non-Responder and Responder groups (*p* = 0.125). Importantly, a similar analysis on perceptual sensitivity (*d*′) also showed no significant differences between the groups (*p* = 0.239). While the first result was not expected, the latter support our hypothesis that stress group has no effect on perceptual performance.
