# Corrigendum: Biological Stress Reactivity and Introspective Sensitivity: An Exploratory Study

**DOI:** 10.3389/fpsyg.2020.00780

**Published:** 2020-04-15

**Authors:** Mauricio Barrientos, Leonel Tapia, Jaime R. Silva, Gabriel Reyes

**Affiliations:** ^1^Centro de Apego y Regulación Emocional, Facultad de Psicología, Universidad del Desarrollo, Santiago, Chile; ^2^Centro de Estudios en Neurociencia Humana y Neuropsicología, Facultad de Psicología, Universidad Diego Portales, Santiago, Chile

**Keywords:** introspection, biological stress reactivity, TSST, consciousness, cortisol

In the published article, there was an error in affiliation 2. The center “Centro de Estudios en Neurociencia Humana y Neuropsicología” was missing.

The authors apologize for this error and state that this does not change the scientific conclusions of the article in any way. The original article has been updated.

