# Supplementary material for: Biological Stress Reactivity and Introspective Sensitivity: An Exploratory Study
Source: Front Psychol. 2020 Mar 26;11:543. doi: 10.3389/fpsyg.2020.00543 (PMC7135889; doi:10.3389/fpsyg.2020.00543)
Supplement: Supplementary file 1 [file Table_1.DOCX]

**Supplementary Material**

**SM-I (Supplementary Material I): Cortisol Concentration Analysis**

In order to measure the saliva cortisol concentration, we followed the standard procedure. Saliva samples were thawed and centrifuged for 6 minutes at 4000 g. The determination of cortisol concentration was conducted using a cortisol ELISA kit (R&D Systems, Inc., Minneapolis, MN, USA) according to the manufacturer’s specifications. Briefly, the ELISA technique (enzyme-linked immunosorbent assay) is based on the competition between the cortisol (antigen) and an enzyme-labeled antigen to bind with an antibody situated on the microplates. We evaluated in duplicate 100 μl of each sample, and the absorbance was measured in a microplate reader set to 450 nm (Stat Fax 2100, Awareness Technologies, MA, USA). The cortisol concentration in each of the samples was obtained from the standard curve created in a concentration range between 0–10 ng/mL.

**SM-II (Supplementary Material II): iRT vs. RT**


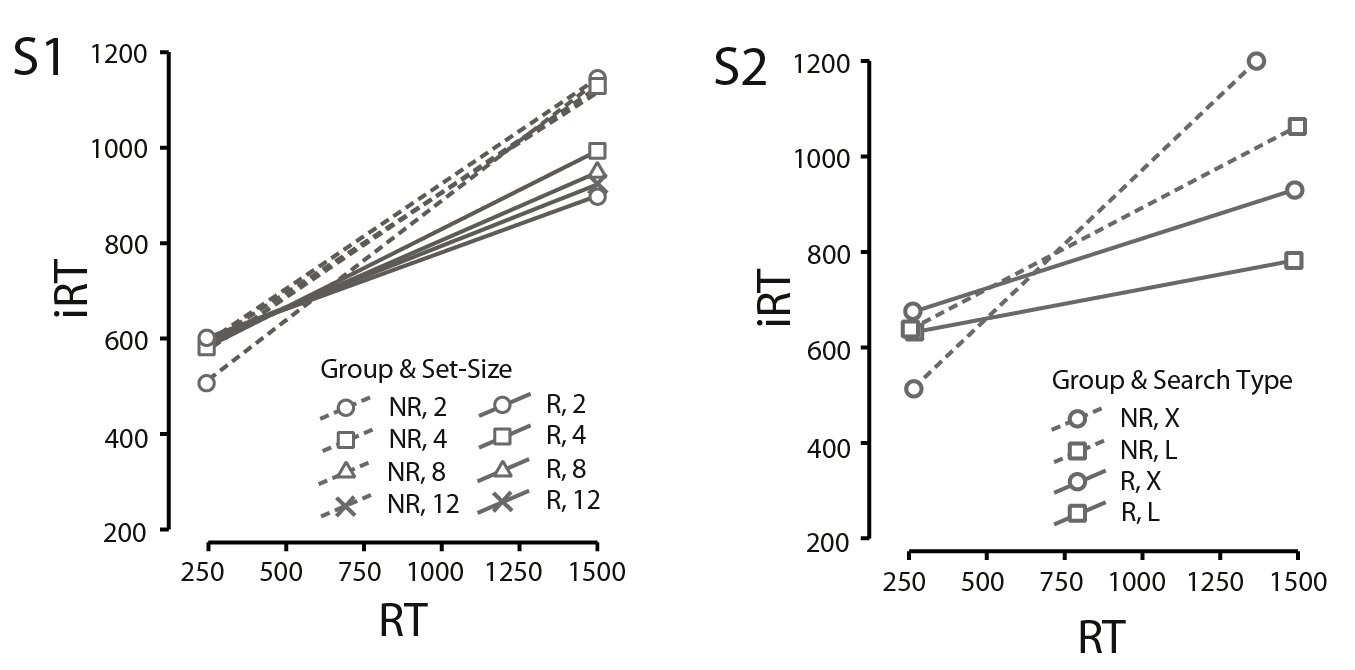


**Figure SM-II:** (S1) Regression of iRT on RT by conditions Group (R, NR) and Set-Size (2, 4, 8 and 12). (S2) Regression of iRT on RT by conditions Group (R, NR) and Search Type (X, L).
